# Chimeric antigen receptor T cells engineered to secrete CD40 agonist antibodies enhance antitumor efficacy

**DOI:** 10.1186/s12967-021-02750-4

**Published:** 2021-02-18

**Authors:** Yajun Zhang, Pei Wang, Tengjiao Wang, Yuan Fang, Yongmei Ding, Qijun Qian

**Affiliations:** 1 Department of Biotherapy, Eastern Hepatobiliary Surgery Hospital, Navy Medical University, Shanghai, 201805 China; 2 Shanghai Engineering Research Center for Cell Therapy, Shanghai, 201805 China; 3 Department of Bioinformatics, Institute of Translational Medicine, Navy Medical University, Shanghai, 201805 China; 4 College of Life Sciences and Medicine, Zhejiang Sci-Tech University, Hangzhou, 310018 Zhejiang China; 5 Department of Medical Oncology, Shanghai Mengchao Cancer Hospital, Shanghai, 201805 China; 6 Shanghai University Cell Therapy Innovation Research Institute, Shanghai, 201805 China

**Keywords:** Chimeric antigen receptor, Anti-CD40 antibody, Costimulatory signal, piggyBac transposon, Solid tumor

## Abstract

**Background:**

Although chimeric antigen receptor (CAR)-T cell therapy has been remarkably successful for haematological malignancies, its efficacy against solid tumors is limited. The combination of CAR-T cell therapy with immune checkpoint inhibitors (CPIs), such as PD-1, PD-L1, and CTLA-4 antibodies, is a promising strategy for enhancing the antitumor efficacy of CAR-T cells. However, because most patients acquire resistance to CPIs, investigating other strategies is necessary to further improve the antitumor efficacy of CAR-T cell therapy for solid tumors. Recently, CD40 agonist antibodies showed potential antitumor efficacy by activating the CD40 pathway.

**Results:**

Based on the piggyBac transposon system, rather than the widely used viral vectors, we constructed a meso3-CD40 CAR-T targeting region III of mesothelin (MSLN) that possessed the ability to secrete anti-CD40 antibodies. Compared with meso3 CAR-T cells, which did not secrete the anti-CD40 antibody, meso3-CD40 CAR-T cells secreted more cytokines and had a relatively higher proportion of central memory T (T_CM_) cells after stimulation by the target antigen. In addition, compared with meso3 CAR-T cells, meso3-CD40 CAR-T cells had a more powerful cytotoxic effect on target cells at a relatively low effector-to-target ratio. More importantly, we demonstrated that the antitumor activity of meso3-CD40 CAR-T cells was enhanced in a human ovarian cancer xenograft model in vivo.

**Conclusions:**

In conclusion, these results highlight anti-CD40-secreting CAR-T cells generated by nonviral vectors as a potential clinical strategy for improving the efficacy of CAR-T cell therapies.

## Background

Recent developments have demonstrated that chimeric antigen receptor (CAR)-T cell therapy can achieve a durable antitumor response in patients with refractory or relapsed B cell malignancies [1, 2]. However, CAR-T cell treatment has been largely ineffective in patients with advanced solid tumors [3]. A critical factor contributing to the limitation of CAR-T cell therapy in patients with advanced solid malignancies may be the immunosuppressive tumor microenvironment. Recently, strategies that utilize an inhibitory checkpoint blockade to modulate the microenvironment have been shown to enhance the efficacy of CAR-T cells in some patients with haematologic malignancies [4]. However, no significant benefit of PD-1 blockade was found in an early-phase trial of a GD2-CAR for the treatment of neuroblastoma [5], thus illustrating the need to consider approaches that employ other immune mechanisms to improve the efficacy of CAR-T cell immunotherapy for solid cancers.

In addition to inhibitory immune regulators, stimulatory checkpoint pathways are promising targets for cancer immunotherapy. CD40, a member of the TNF receptor superfamily, is expressed primarily in antigen-presenting cells (APCs), including dendritic cells (DCs), and its activation in DCs by CD40L expressed on CD4^+^ T cells has been shown to be critical for promotion of the immune response and antitumor activation of CD8^+^ T cells by DCs [6–8]; thus, CD4 indirectly aids CD8^+^ T cells. However, other studies have shown that activated CD8^+^ T cells also express CD40 and that these CD40^+^ CD8^+^ T cells can receive CD4 help directly via CD40, and this direct CD4^+^-CD8^+^ T cell interaction can enhance cell division and cytokine secretion in CD8^+^ T cells [9].

Although the mechanisms underlying the CD40 pathway are not fully understood and are being actively investigated, studies leveraging this pathway for potent antitumor activity have shown definite encouraging efficacy. These studies include the use of CD40L [10, 11] but most notably the use of anti-CD40 antibodies [12], especially the combination of anti-CD40 antibodies with other therapies [13, 14], because the antitumor activity of an anti-CD40 antibody as a single agent is relatively limited [15]. Furthermore, activating the CD40 pathway in CAR-T cell therapy through various strategies has enhanced the antitumor activity of CAR-T cells. These strategies include engineering CAR-T cells to constitutively express CD40L [16, 17], introducing the MyD88 and CD40 signalling domains into CAR-T cells [18], and administering a bispecific antibody targeting c-Myc and CD40 [19]. In addition to these studies, we are very interested in investigating whether CAR-T cells engineered to secrete anti-CD40 antibodies exhibit an enhanced antitumor activity, as similar approaches regarding the engineering of CAR-T cells to secrete anti-PD-1 antibodies have demonstrated efficacy in improving their antitumor activity [20, 21].

Our previous study demonstrated that modified CAR-T cells targeting the membrane proximal (region III) epitope of mesothelin (MSLN) exhibited strong antitumor activity against various solid tumors [22, 23]. Given this, CAR-T cells targeting region III of MSLN were further engineered with the ability to secrete anti-CD40 antibodies to potentially exhibit an even more powerful antitumor efficacy. The expensive costs and potential presence of replication-competent viruses in the final cell products of viral vectors have impeded their vast implementation in the industry [24]. In this study, we manufactured CAR-T cells using the piggyBac transposon system to overcome some of these obstacles. More importantly, compared with treatment with meso3 CAR-T cells, the manufactured meso3-CD40 CAR-T cells secreting the anti-CD40 antibody used in this study seemed to be a more effective therapeutic strategy for tumors. Overall, in terms of cost and efficacy, generating CAR-T cells that secrete anti-CD40 antibodies by exploiting the piggyBac transposon method appears promising for cancer treatment.

## Materials and methods

### Cells

This study was conducted according to the ethical guidelines of Navy Medical University. Human peripheral blood mononuclear cells (PBMCs) were obtained from healthy donors from Shanghai Aoneng Biotechology Co., Ltd. (Shanghai, China) and cryopreserved in our laboratory. The SKOV-3 cell line was obtained from the Cell Bank of the Chinese Academy of Sciences (Shanghai, China), and SKOV-3-luc was established in our laboratory as described previously [22].

### Plasmid construction

The plasmid encoding the anti-CD40 antibody, pS338B-CD40, was constructed based on our pS338B vector and consisted of the following components: an EcoRI site, a signal peptide derived from the human immunoglobulin kappa chain, the CD40 scFv heavy chain variable region, a (G4S)3 linker, the CD40 scFv light chain variable region, the human IgG4 Fc (EQ) region, and a SalI site. The plasmid encoding meso3 CAR, pNB338B-meso3 CAR, was constructed based on the pNB338B vector. Meso3 CAR comprises the scFv of the anti-MSLN antibody, the CD28 transmembrane domain, the CD28 intracellular co-stimulatory signaling domain and CD3ζ. The mock plasmid was acquired by replacing the CAR gene of the pNB338B-meso3 CAR plasmid using an empty multiple cloning site (MCS).

### CAR-T cell manufacturing and expansion

The manufacture and expansion of meso3 CAR-T cells has been described previously[22]. To generate meso3-CD40 CAR-T cells, T cells were coelectroporated with a pNB338B-meso3 CAR plasmid and a pS338B-CD40 plasmid using an electroporator (Lonza) and the Amaxa® Human T Cell Nucleofector® Kit (Lonza). After transfection, CAR-T cells were cultured in AIM-V medium containing 2% FBS and then transferred to six-well plates coated with the rhMSLN antigen (5 μg/mL) and anti-CD28 (5 μg/mL) antibody after 4 h. The cells were stimulated by coated plates for 3–4 days in medium containing 200 U/mL recombinant human interleukin-2 (rhIL-2), and the activated CAR-T cells were then cultured in 2% FBS-AIM-V medium containing 100 U/mL rhIL-2 for another 5–10 days. Mock T cells were generated by electroporation with the mock plasmid and then stimulated with anti-CD3 (5 μg/mL) and anti-CD28 (5 μg/mL) antibodies. Thereafter, the culture method for mock T cells was the same as that used for meso3 CAR-T cells. Subsequent experiments were typically conducted 10 days after electroporation.

### ELISA

The anti-CD40 antibody concentration in the CAR-T cell supernatant was determined by ELISA. A total of 1 × 10^6^ CAR-T cells were seeded in six-well plates coated with the rhMSLN antigen, and the supernatant of CAR-T cells was then collected after 24 h or 72 h.

A 96-well ELISA microplate was coated with 1 μg/ml rhCD40 (ACROBiosystems) at 4 °C overnight. After the microplate was sealed with 1% BSA for 2 h at 37 °C, the collected supernatant was added to the well and incubated for 1 h at 37 °C. After washing the wells three times, mouse anti-human IgG4 (HRP) (Abcam) was added to the well at a dilution of 1:30,000 and incubated for 30 min at 37 °C. After washing the wells three times, 3,3′,5,5′-tetramethylbenzidine (TMB) was added to develop the color. All of the experiments were performed in triplicate.

### Cytokine release assays

Cytokine release assays were performed by coculturing 1 × 10^6^ CAR-T cells with immobilized rhMSLN antigen in six-well plates. After 24 h, the supernatants were assayed for the presence of all cytokines using the Cytometric Bead Array according to the manufacturer’s instructions (BD Biosciences). The values represent the mean of the triplicate wells.

### Flow cytometry

We detected the expression of CD40 in the different subsets of T cells before or after stimulation for different times. The antibodies used included BB515 mouse anti-human CD40 (BD Horizon), APC-Cy7 mouse anti-human CD8 (BD Pharmingen), PerCP-Cy5.5 Mouse anti-human CD4 (BD Pharmingen) and BV510 mouse anti-human CD3 (BD Horizon) antibodies were used. On the 10th day after electroporation, CAR was expressed on the surface of the cells, and the cells were stained with biotin-conjugated rhMSLN antigen (ACROBiosystems) and PE-conjugated streptavidin (BD Biosciences). The following monoclonal antibodies were used for the phenotypic analysis: PE-Cy5 anti-human CD45RO (BioLegend), PE anti-human CD62L (BioLegend), FITC anti-human CD197 (BioLegend), PE-Cy5 anti-human CD25 (BioLegend), PC5 anti-human CD69 (BioLegend), and PE-CY5 anti-human CD107α (BD Biosciences). The surface expression of MSLN on SKOV-3 cells was detected using a primary self-synthesized anti-MSLN antibody with Fc fragments and a PE anti-human IgG Fc secondary antibody (BioLegend). The acquisition and analysis were performed using a Navios™ flow cytometer (Beckman Coulter, USA) and Kaluza Analyse software.

### Real-time cytotoxicity assay (RTCA)

Real-time cytotoxicity assays were performed as described previously [22]. Briefly, targeted cells were seeded in 16-well E-plates at a density of 10,000 cells per well. After 20–24 h, CAR-T cells were added at effector-to-target ratios (E:T) of 1:2 and 1:4, while mock T cells were added at an E:T of 2:1.

### In vivo experiments

All of the animal experiments were approved by the Institutional Animal Care and Use Committee (IACUC) of Shanghai Bioduro Biologics Co., Ltd. Six- to eight-week-old female NOD/SCID/IL2Rγ−/− (NSG) mice were purchased from Shanghai Bioduro Biologics Co., Ltd. and raised under specific pathogen-free (SPF) conditions. The mice were euthanized after exhibiting moribund features, such as a large tumor burden, obvious weight loss, hunched posture, and lethargy. Xenograft tumors were established by the subcutaneous injection of 1 × 10^7^ SKOV-3-luc cells in the presence of a 50% solution of Matrigel (BD Biosciences) in phosphate buffered saline (PBS). Eight days post inoculation, mice bearing established tumors were treated with intravenous injections of 5 × 10^6^ meso3 CAR-T or meso3-CD40 CAR-T cells, and the bioluminescence was measured using serial imaging on a Xenogen IVIS Spectrum System (Life Technologies).

### Statistical analysis

The data are presented as the means ± standard errors of the means (SEM) or as medians with ranges, as stated in the figure legends. Student’s t test or the Mann–Whitney test was used to compare to selected groups. Statistical significance was defined at P < 0.05, and all statistical analyses were performed using Prism software version 6.0 (GraphPad).

## Results and discussion

**Fig. 1:**
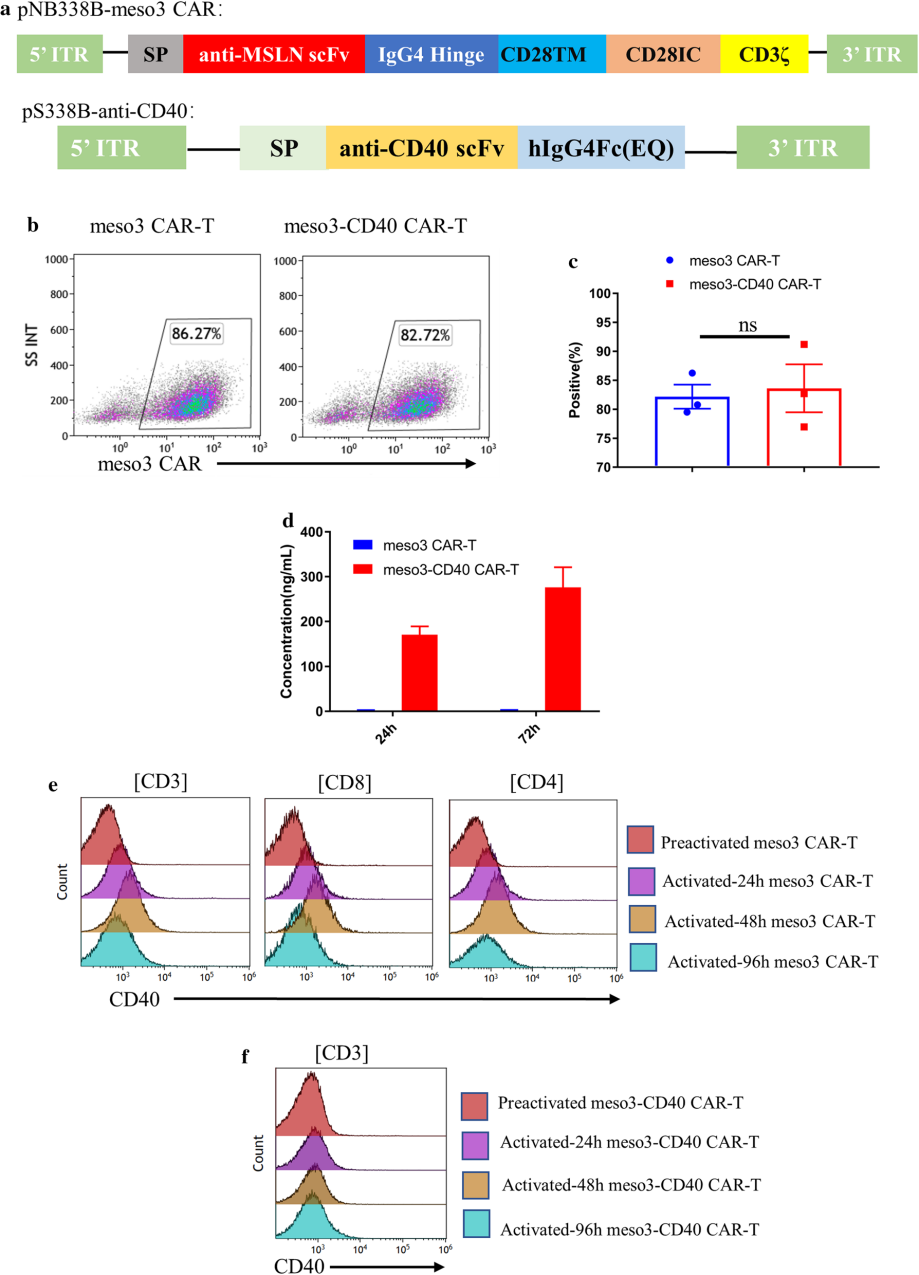
Construction and characterization of meso3 CAR-T and meso3-CD40 CAR-T cells. **a** Schematic representation of the plasmid vector encoding the anti-CD40 antibody and meso3 CAR. **b** Ten days after electroporation, the positive ratio of meso3 CAR in meso3 CAR-T and meso3-CD40 CAR-T cells was detected by flow cytometry. **c** The summarized statistics of three donors are shown in the bar graphs (n = 3, mean ± SEM, ns, not significant). **d** The anti-CD40 antibody levels in the supernatants of meso3 CAR-T and meso3-CD40 CAR-T cells were determined by ELISA, and the results are shown in the bar graphs (n = 3, mean ± SEM). **e**, **f** CD40 expression in meso3 CAR-T and meso3-CD40 CAR-T cells after stimulation with rhMSLN for different amounts of times

A schematic representation of the plasmid vector encoding the anti-CD40 antibody is shown in Fig. 1a. The scFv sequence of the anti-CD40 antibody was derived from a fully human IgG2 anti-human anti-CD40 antibody clone, 21.4.1 [25]. To prevent the death of potential target cells (CAR-T cells expressing CD40) due to immune effector functions of the anti-CD40 antibody, such as antibody-dependent cell-mediated cytotoxicity (ADCC) and complement-dependent cytotoxicity (CDC), the scFv was fused to a human IgG4 Fc, which has a very limited ability to elicit these effector functions. In addition, to further reduce the effector function of human IgG4 Fc, a human IgG4 variant with two point mutations at L235E and N297Q was generated, which has been demonstrated to further reduce the effector function of the antibody [26].

We previously showed that the piggyBac transposon system is an efficient vector to generate CAR-T cell products [22, 23, 27], and we herein demonstrated that meso3-CD40 CAR-T cells secreting CD40 were successfully manufactured by the coelectroporation of CAR-T cells with two plasmids, pS338B-CD40 and pNB338B-meso3 CAR. Moreover, the introduction of additional anti-CD40 antibody genes did not affect the level of CAR expression on the T cell surface (Fig. 1b, c). Other similar studies have also demonstrated that the introduction of an additional antibody gene in the generation of CAR-T cells does not severely affect the expression of CAR [20, 21].

After generating meso3-CD40 CAR-T cells, the ability of meso3-CD40 CAR-T cells to secrete the anti-CD40 antibody was determined by ELISA. The anti-CD40 antibody concentration in the supernatant of 1 × 10^6^ meso3-CD40 CAR-T cells was approximately 0.17 μg/ml at 24 h and approximately 0.28 μg/ml at 72 h of culture, while no antibody secretion was detected in the meso3 CAR-T cell culture supernatant (Fig. 1d). These results showed that the meso3-CD40 CAR-T cells secreted antibodies continuously and stably and were consistent with the levels of antibodies secreted by CAR-T cells reported in similar studies that used viral vectors to generate antibody-secreting CAR-T cells [20, 21].

A previous study reported the transient expression of CD40 in both CD4^+^ and CD8^+^ T cells after activation [9]. To further demonstrate this, CAR-T cells were activated using rhMSLN antigen coated on plates to investigate the expression pattern of CD40 in various states of activation. After activation, the CD40 expression on the CD4^+^ and CD8^+^ meso3 CAR-T cell subsets increased over time, peaking at 48 h, and then decreased to the basal level at 72 h (Fig. 1e). However, no obvious CD40 expression was detected in the meso3-CD40 CAR-T group in any of the stages (Fig. 1f), which was potentially attributed to the blocking effect of the anti-CD40 antibody in the culture medium. This result was consistent with those of recent studies showing that CAR-T secreting antibodies could bind to the target antigen expressed in T cells [21, 28].

**Fig. 2:**
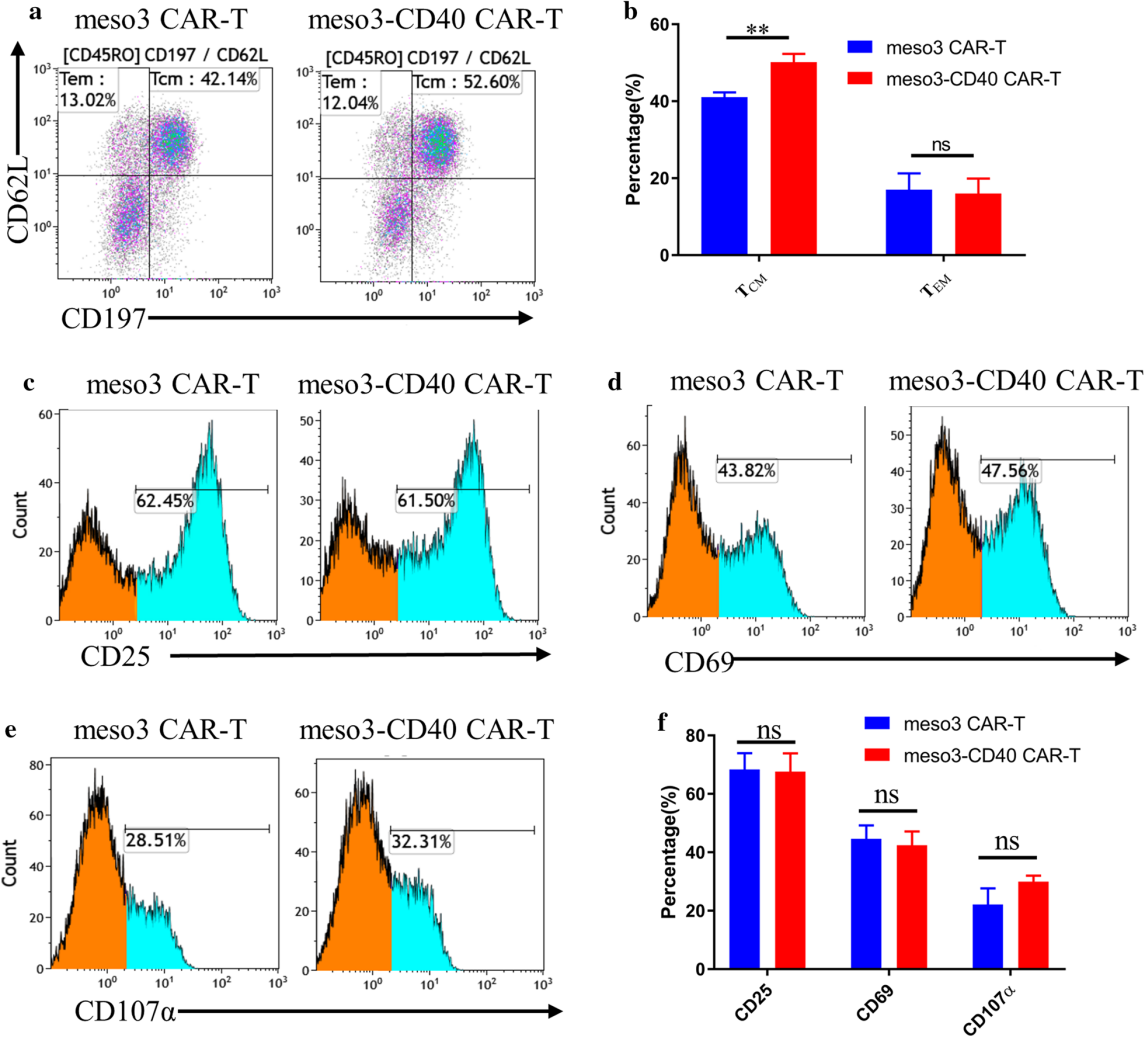
Phenotypic analysis of the meso3 CAR-T and meso3-CD40 CAR-T cell products. **a** The percentages of T_CM_ and T_EM_ in meso3 CAR-T and meso3-CD40 CAR-T cells were detected by flow cytometry. Memory T cells were gated using an anti-human CD197 antibody and an anti-human CD62L antibody from CD45RO^+^ T cells. **b** The summarized statistics regarding the memory T cells from three donors are shown in the bar graphs (n = 3, mean ± SEM, ns, not significant, *P < 0.05; **P < 0.01; ***P < 0.001). **c**–**e** Activation markers on the surface of T cells were detected by flow cytometry upon stimulation with the rhMSLN antigen for 24 h. **f** The summarized statistics of the activation markers on the CAR-T cells from three donors are shown in the bar graphs (n = 3, mean ± SEM, ns, not significant)

CD40-CD40L signaling was previously reported to be essential for the development of memory T cells that depend on fatty acid metabolism for long-term survival [29], and CD40 expression on T cells is fundamental for CD8^+^ T cell memory generation [9]. Therefore, the memory phenotypes of T cells were detected, and the results shown in Fig. 2a, b suggest that no significant differences existed in the proportions of effector memory T cells (T_EM_, CD45RO^+^CD197^−^) between the two groups, while meso3-CD40 CAR-T cells exhibited a higher proportion of central memory T cells (T_CM_, CD45RO^+^CD197^+^CD62L^+^) than meso3 CAR-T cells (50.16 ± 1.2 versus 41.07 ± 0.72%, p = 0.0032). T_EM_ potentially localizes in peripheral lymphoid tissues and possesses a rapid effector function, while T_CM_ tends to localize in secondary lymphoid tissues and maintain T cell memory for a long period. Given the close correlation of T_CM_ with the prevention of long-term tumor recurrence, a high proportion of T_CM_ may enhance antitumor effects in vivo [30, 31].

CD25, CD69, and CD107a are known activation markers of lymphocytes and play pivotal roles in immune responses. To characterize the CAR-T cells, the activation markers on CAR-T cell surfaces were detected upon stimulation with rhMSLN antigen-coated plates for 24 h. After stimulation for 24 h, all of the activation markers (CD25, CD69, and CD107α) on the meso3-CD40 CAR-T cells were expressed at levels equivalent to those on meso3 CAR-T cells (CD25: 67.7 ± 3.6 versus 68.4 ± 3.2%, p = 0.8873; CD69: 42.4 ± 2.7 versus 44.6 ± 2.7%, p = 0.5965; CD107α: 22.1 ± 3.2 versus 29.9 ± 1.2%, p = 0.0832) (Fig. 2c–f), demonstrating that the meso3-CD40 CAR-T and meso3 CAR-T cells were sensitive to the MSLN antigen and effectively activated by the MSLN antigen and thus suggesting their potential for eradicating tumor cells.

**Fig. 3:**
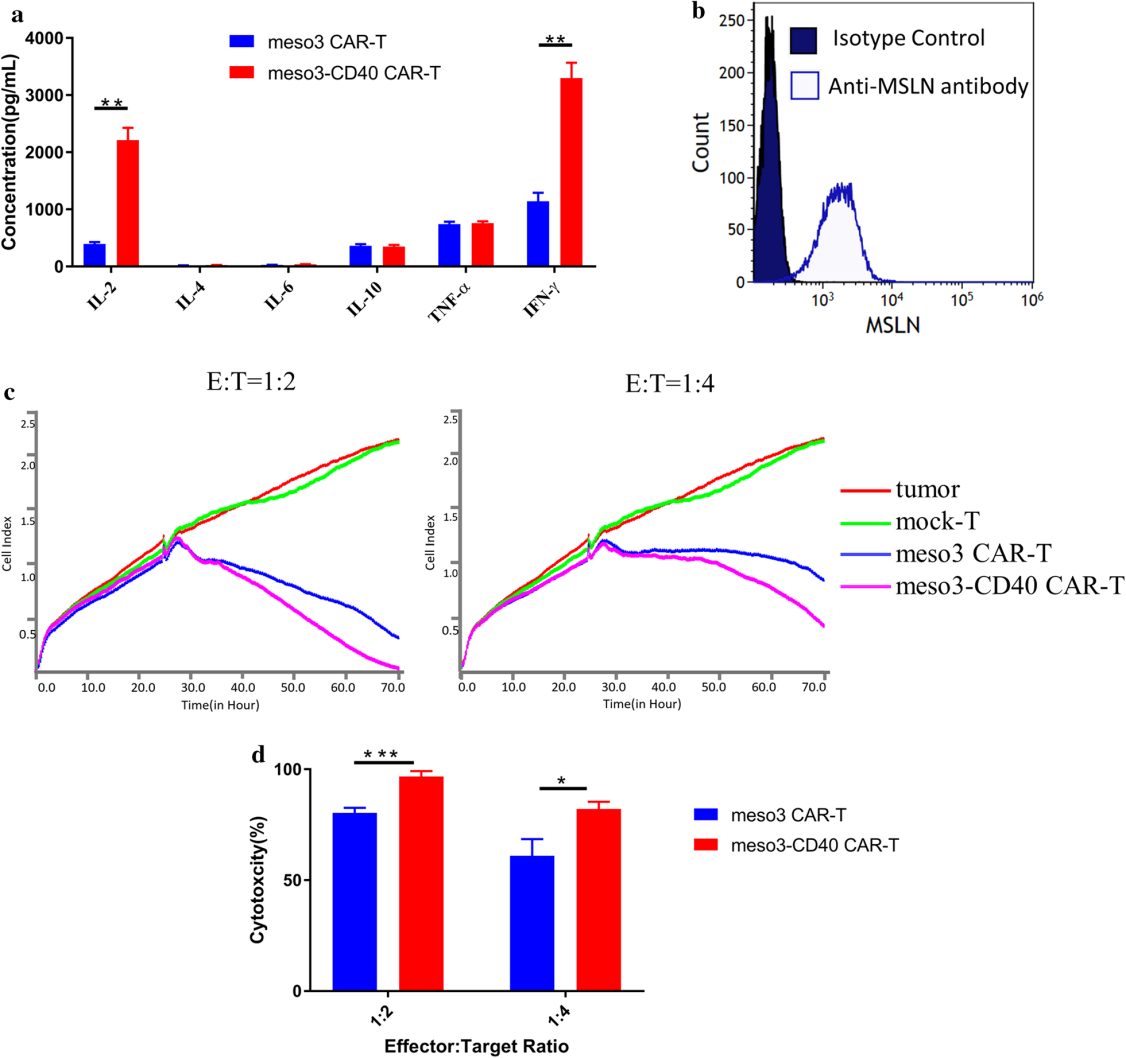
Cytokine secretion and cytotoxic activity of the meso3 CAR-T and meso3-CD40 CAR-T cells. **a** Cytokine secretion from meso3 CAR-T and meso3-CD40 CAR-T cells was determined by a cytometric bead array after costimulation with the rhMSLN antigen. **b** The MSLN expression level in SKOV-3 cells was validated by flow cytometry. **c** The cytotoxicities of the meso3 CAR-T and meso3-CD40 CAR-T cells against SKOV3 cells were assessed using the RTCA system at low effector-to-target ratios (E:T = 1:2 and 1:4), and the mock T cells group was used as a negative control. **d** Quantification of the data of the specific cytotoxicities of CAR T cells against SKOV-3 cells at different E:T ratios (n = 3, mean ± SEM, ns, not significant, *P < 0.05; **P < 0.01; ***P < 0.001)

In addition, we next determined the cytokine secretion levels after the stimulation of CAR-T cells with the target antigen, revealing that IL-2 was expressed at significantly higher levels in the meso3-CD40 CAR-T group than in the meso3 CAR-T group (2244 ± 213.7 versus 390.7 ± 31.51 pg/ml, p = 0.001). In addition, the concentration of IFN-γ was significantly higher in the meso3-CD40 CAR-T group than in the meso3 CAR-T group (3297 ± 272.2 versus 1143 ± 149.8 pg/ml, p = 0.0023), while the differences in the levels of the other cytokines tested were not statistically significant between the two groups (Fig. 3a). A previous study showed that CD40 expression on T cells was essential for high levels of cytokine secretion [9], and we herein demonstrated that anti-CD40 antibody production further enhanced cytokine secretion. Given that IFN-γ is closely correlated with efficacy, it is reasonable to speculate that the antitumor efficacy of meso3-CD40 CAR-T cells is better than that of meso3 CAR-T cells. To validate this in vitro, SKOV-3 (human ovarian cancer) cells were used as the target because of their high MSLN antigen expression levels (Fig. 3b). Indeed, as shown in Fig. 3c, d, although the CAR-T cells of both groups showed potent cytotoxic activity against SKOV-3 cells, that of the meso3-CD40 CAR-T cells was stronger than the meso3 CAR-T cell activity in vitro (E:T = 1:2, 96.8 ± 1.4 versus 80.3 ± 1.3%, p = 0.0009; E:T = 1:4, 82.2 ± 1.8 versus 61.0 ± 4.3%, p = 0.011), while the mock T cells exhibited no obvious cytotoxicity against SKOV-3 cells.

In our previous studies, the cytotoxic activity of meso3 CAR-T cells was analysed at E:T ratios of 4:1 to 1:1. Considering their potent cytotoxic activities, observing the differences in their cytotoxic activities of meso3 CAR-T and meso3-CD40 CAR-T cells can be difficult because they can lyse target cells in a short time. In this study, we adopted a relatively low E:T ratio to allow CAR-T cells to lyse the target cell more gently and thereby observed the benefit of the anti-CD40 antibody.

**Fig. 4:**
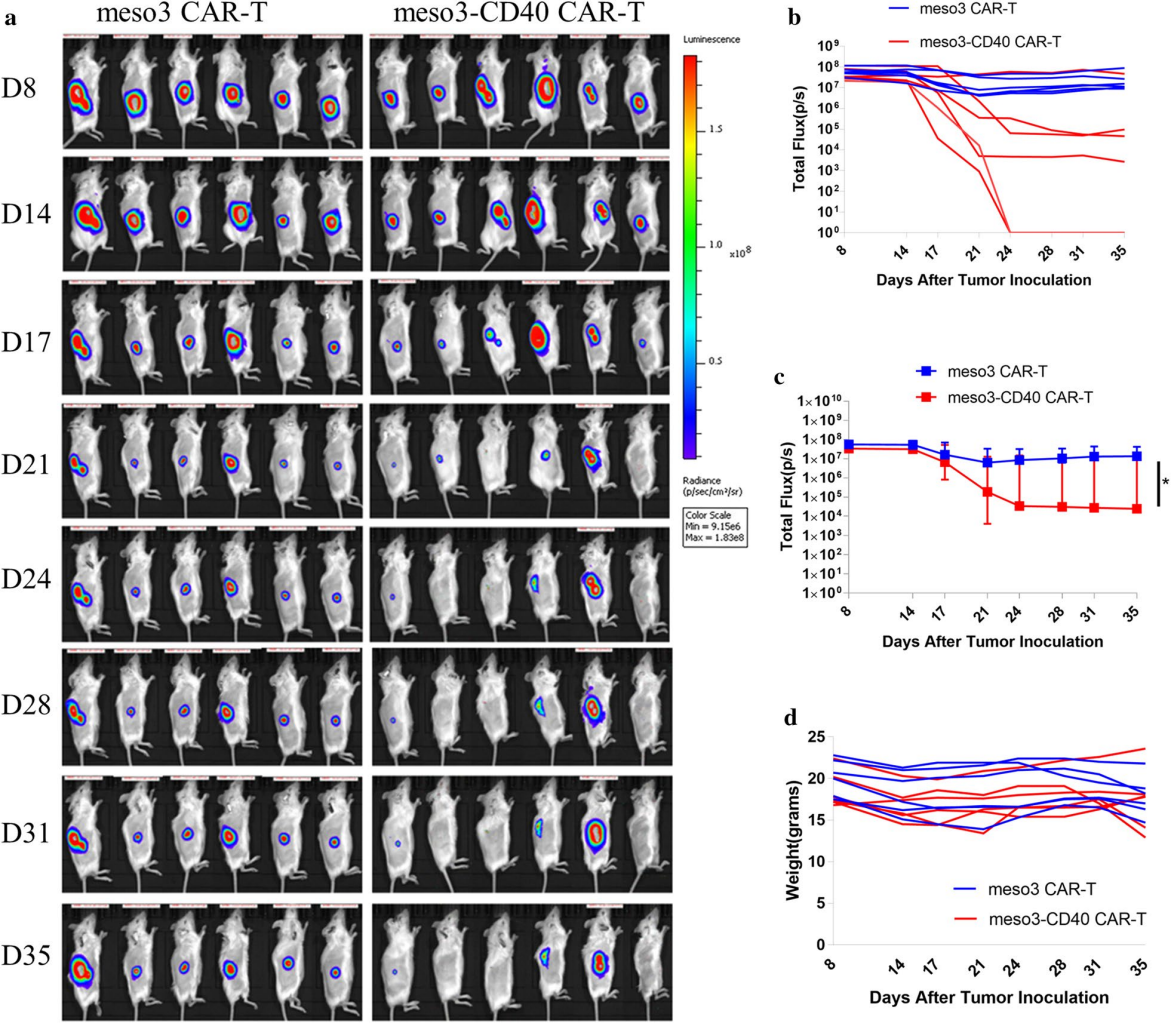
CAR-T cells that secrete the anti-CD40 antibody have enhanced antitumor function in vivo. **a** Bioluminescence signals from SKOV-3-luc cancer model mice during treatment. **b** The quantified fluorescence intensities of SKOV-3-luc for individual mice are shown in the line graphs. **c** The fluorescence intensity of SKOV-3-luc was analysed (n = 6, medians with range, Mann–Whitney test, *P < 0.05). **d** The body weights of individual mice were recorded and are shown in the line graphs

Meso3-CD40 CAR-T cells had more potent antitumor activity against MSLN-positive cells than meso3 CAR-T cells in vitro, even at a low E:T ratio, and we next investigated whether similar effects were observable in vivo. Xenograft tumor models were established by subcutaneously inoculating 1 × 10^7^ SKOV-3-luc cells into mice. Eight days post inoculation, the mice were treated with intravenous injections of 5 × 10^6^ meso3 CAR-T or meso3-CD40 CAR-T cells, and in vivo imaging intuitively reflected that both the meso3 CAR-T and meso3-CD40 CAR-T cells effectively inhibited tumor growth in vivo. Encouragingly, the meso3-CD40 CAR-T cells exerted faster and stronger effects than the meso3 CAR-T cells (Fig. 4a). Consistent with the in vivo cancer images, the fluorescence intensity of the group treated with meso3-CD40 CAR-T cells attenuated more quickly (Fig. 4b) and was significantly lower than that of the meso3 CAR-T group (Fig. 4c, p = 0.039, Mann–Whitney test). In addition, the mouse weights were measured continuously and were not obviously altered after the treatments (Fig. 4d). Overall, these results validated that meso3-CD40 CAR-T cells exhibited better antitumor activity than meso3 CAR-T cells in a xenograft mouse model.

Kuhn et al. demonstrated that a more potent antitumor response can be achieved by further engineering CAR-T cells to constitutively express CD40L, and this effect is dependent on host CD40 expression and DC activation [17]. However, the enhanced antitumor activity of meso3-CD40 CAR-T cells compared to meso3 CAR-T cells observed herein both in vitro and in vivo may be attributed to only the improved CAR-T cell function, as there were no DCs in the in vitro RTCA assay and the NSG mice used in the study were severely immune deficient and had only defective DCs and macrophages. Additionally, the anti-human anti-CD40 antibody used in our study does not cross-react with murine CD40 [25]. Thus, it is reasonable to speculate that CAR-T cells secreting the anti-CD40 antibody will induce a more potent antitumor effect in a syngeneic immunocompetent mouse model, as the anti-CD40 antibody not only enhances CAR-T cell response independently of DCs but also aids in antitumor responses via DCs expressing CD40. However, this hypothesis needs to be investigated in a syngeneic immunocompetent model.

To further enhance the antitumor activity of CAR-T cells in solid tumors, modulating the immunosuppressive tumor microenvironment may be a promising approach. Previous reports have demonstrated that CAR-T cells engineered to secrete anti-PD-1 antibodies at the tumor site have an antitumor efficacy that is similar to or even better than that of combination therapy with CAR-T cells and CPIs [21]. In our study, we demonstrated that a more potent antitumor response was achievable by engineering CAR-T cells to secrete anti-CD40 antibodies. However, one potential limitation of the study is that we did not investigate whether meso3-CD40 CAR-T cells had a similar efficacy to meso3 CAR-T cells used together with a recombinant anti-CD40 antibody, which would be especially interesting in a syngeneic immunocompetent mouse model.

Another potential limitation is the uncontrolled antibody secretion from CAR-T cells, which may be a potential safety concern. Previously, Rafiq et al. demonstrated that the anti-PD-1 scFv secreted by CAR-T cells was detectable in only the local tumor microenvironment, whereas systemically administered anti-PD-1 antibodies could circulate out of the tumor area [21]. Likewise, the secreted anti-CD40 antibodies from meso3-CD40 CAR-T cells were not found systemically in our mouse model (data not shown). However, Li et al. showed that the anti-PD-1 scFv secreted by CAR-T cells was also detectable in the sera, although at a relatively low concentration [20]. To further ensure that the antibodies secreted by CAR-T cells induce fewer systemic toxicities, this potential safety concern is worthy of investigation. Encouragingly, we recently successfully designed a T cell-specific and highly active artificial chimeric promoter to control the secretion of anti-PD-1 antibodies at the tumor site [28]. Studies using this chimeric promoter to control the secretion of anti-CD40 antibodies from CAR-T cells in a syngeneic immunocompetent mouse model are currently underway to explore the potential applicability of anti-CD40 antibody-secreting CAR-T cells and thus further improve the antitumor activity of this therapy for solid tumors.

## Conclusion

Meso3-CD40 CAR-T cells secreted more cytokines and had a higher proportion of central memory T cells than meso3 CAR-T cells after stimulation with the target antigen. Notably, meso3-CD40 CAR-T cells also had a more potent antitumor response in vitro and in vivo. It is worth mentioning that the use of the nonviral piggyBac transposon in this study provides strong competitiveness for industrialization development due to its considerable cost savings. Therefore, CAR-T cells that are piggyBac-engineered to secrete anti-CD40 antibody could be an effective and low-cost therapeutic strategy to further improve the efficacy of CAR-T cell therapy for solid tumors.

## Data Availability

All the authors confirm that the data supporting the findings of this study are available within the article and its supplementary materials.

## Abbreviations

CAR-T: Chimeric antigen receptor T-cell
MSLN: Mesothelin
IACUC: Institutional Animal Care and Use Committee
APCs: Antigen presenting cells
PBMC: Peripheral blood mononuclear cells
ADCC: Antibody-dependent cell-mediated cytotoxicity
CDC: Complement dependent cytotoxicity
T_EM_: Effector memory T
T_CM_: Central memory T
CPIs: Checkpoint inhibitors

## References

[1] Tran E, Longo DL, Urba WJ (2017). A milestone for CAR T cells. N Engl J Med.

[2] Halim L, Maher J (2020). CAR T-cell immunotherapy of B-cell malignancy: the story so far. Ther Adv Vaccines Immunother.

[3] Newick K, O'Brien S, Moon E, Albelda SM (2017). CAR T cell therapy for solid tumors. Annu Rev Med.

[4] Chong EA, Melenhorst JJ, Lacey SF, Ambrose DE, Gonzalez V, Levine BL, June CH, Schuster SJ (2017). PD-1 blockade modulates chimeric antigen receptor (CAR)-modified T cells: refueling the CAR. Blood.

[5] Heczey A, Louis CU, Savoldo B, Dakhova O, Durett A, Grilley B, Liu H, Wu MF, Mei Z, Gee A (2017). CAR T cells administered in combination with lymphodepletion and PD-1 inhibition to patients with neuroblastoma. Mol Ther.

[6] Ridge JP, Di Rosa F, Matzinger P (1998). A conditioned dendritic cell can be a temporal bridge between a CD4+ T-helper and a T-killer cell. Nature.

[7] French RR, Chan HT, Tutt AL, Glennie MJ (1999). CD40 antibody evokes a cytotoxic T-cell response that eradicates lymphoma and bypasses T-cell help. Nat Med.

[8] Sotomayor EM, Borrello I, Tubb E, Rattis FM, Bien H, Lu Z, Fein S, Schoenberger S, Levitsky HI (1999). Conversion of tumor-specific CD4+ T-cell tolerance to T-cell priming through in vivo ligation of CD40. Nat Med.

[9] Bourgeois C, Rocha B, Tanchot C (2002). A role for CD40 expression on CD8+ T cells in the generation of CD8+ T cell memory. Science.

[10] Vonderheide RH, Dutcher JP, Anderson JE, Eckhardt SG, Stephans KF, Razvillas B, Garl S, Butine MD, Perry VP, Armitage RJ (2001). Phase I study of recombinant human CD40 ligand in cancer patients. J Clin Oncol.

[11] Merz C, Sykora J, Marschall V, Richards DM, Heinonen K, Redondo Muller M, Thiemann M, Schnyder T, Fricke H, Hill O, Gieffers C (2018). The hexavalent CD40 agonist HERA-CD40L induces T-cell-mediated antitumor immune response through activation of antigen-presenting cells. J Immunother.

[12] Vonderheide RH (2020). CD40 agonist antibodies in cancer immunotherapy. Annu Rev Med.

[13] Beatty GL, Torigian DA, Chiorean EG, Saboury B, Brothers A, Alavi A, Troxel AB, Sun W, Teitelbaum UR, Vonderheide RH, O'Dwyer PJ (2013). A phase I study of an agonist CD40 monoclonal antibody (CP-870,893) in combination with gemcitabine in patients with advanced pancreatic ductal adenocarcinoma. Clin Cancer Res.

[14] Nowak AK, Cook AM, McDonnell AM, Millward MJ, Creaney J, Francis RJ, Hasani A, Segal A, Musk AW, Turlach BA (2015). A phase 1b clinical trial of the CD40-activating antibody CP-870,893 in combination with cisplatin and pemetrexed in malignant pleural mesothelioma. Ann Oncol.

[15] Beatty GL, Chiorean EG, Fishman MP, Saboury B, Teitelbaum UR, Sun W, Huhn RD, Song W, Li D, Sharp LL (2011). CD40 agonists alter tumor stroma and show efficacy against pancreatic carcinoma in mice and humans. Science.

[16] Curran KJ, Seinstra BA, Nikhamin Y, Yeh R, Usachenko Y, van Leeuwen DG, Purdon T, Pegram HJ, Brentjens RJ (2015). Enhancing antitumor efficacy of chimeric antigen receptor T cells through constitutive CD40L expression. Mol Ther.

[17] Kuhn NF, Purdon TJ, van Leeuwen DG, Lopez AV, Curran KJ, Daniyan AF, Brentjens RJ (2019). CD40 ligand-modified chimeric antigen receptor T cells enhance antitumor function by eliciting an endogenous antitumor response. Cancer Cell.

[18] Mata M, Gerken C, Nguyen P, Krenciute G, Spencer DM, Gottschalk S (2017). Inducible activation of MyD88 and CD40 in CAR T cells results in controllable and potent antitumor activity in preclinical solid tumor models. Cancer Discov.

[19] von Scheidt B, Wang M, Oliver AJ, Chan JD, Jana MK, Ali AI, Clow F, Fraser JD, Quinn KM, Darcy PK (2019). Enterotoxins can support CAR T cells against solid tumors. Proc Natl Acad Sci USA.

[20] Li S, Siriwon N, Zhang X, Yang S, Jin T, He F, Kim YJ, Mac J, Lu Z, Wang S (2017). Enhanced cancer immunotherapy by chimeric antigen receptor-modified T cells engineered to secrete checkpoint inhibitors. Clin Cancer Res.

[21] Rafiq S, Yeku OO, Jackson HJ, Purdon TJ, van Leeuwen DG, Drakes DJ, Song M, Miele MM, Li Z, Wang P (2018). Targeted delivery of a PD-1-blocking scFv by CAR-T cells enhances anti-tumor efficacy in vivo. Nat Biotechnol.

[22] Zhang Z, Jiang D, Yang H, He Z, Liu X, Qin W, Li L, Wang C, Li Y, Li H (2019). Modified CAR T cells targeting membrane-proximal epitope of mesothelin enhances the antitumor function against large solid tumor. Cell Death Dis.

[23] He J, Zhang Z, Lv S, Liu X, Cui L, Jiang D, Zhang Q, Li L, Qin W, Jin H, Qian Q (2018). Engineered CAR T cells targeting mesothelin by piggyBac transposon system for the treatment of pancreatic cancer. Cell Immunol.

[24] Ramanayake S, Bilmon I, Bishop D, Dubosq MC, Blyth E, Clancy L, Gottlieb D, Micklethwaite K (2015). Low-cost generation of Good Manufacturing Practice-grade CD19-specific chimeric antigen receptor-expressing T cells using piggyBac gene transfer and patient-derived materials. Cytotherapy.

[25] Gladue RP, Paradis T, Cole SH, Donovan C, Nelson R, Alpert R, Gardner J, Natoli E, Elliott E, Shepard R, Bedian V (2011). The CD40 agonist antibody CP-870,893 enhances dendritic cell and B-cell activity and promotes anti-tumor efficacy in SCID-hu mice. Cancer Immunol Immunother.

[26] Wang X, Mathieu M, Brezski RJ (2018). IgG Fc engineering to modulate antibody effector functions. Protein Cell.

[27] Wang P, Qin W, Liu T, Jiang D, Cui L, Liu X, Fang Y, Tang X, Jin H, Qian Q (2020). PiggyBac-engineered T cells expressing a glypican-3-specific chimeric antigen receptor show potent activities against hepatocellular carcinoma. Immunobiology.

[28] Fang Y, Zhang Y, Guo C, Chen C, Gao H, Zhou X, Liu T, Qian Q (2020). Safety and efficacy of an immune cell-specific chimeric promoter in regulating anti-PD-1 antibody expression in CAR T cells. Mol Ther Methods Clin Dev.

[29] Ara A, Ahmed KA, Xiang J (2018). Multiple effects of CD40-CD40L axis in immunity against infection and cancer. Immunotargets Ther.

[30] Rolle CE, Carrio R, Malek TR (2008). Modeling the CD8+ T effector to memory transition in adoptive T-cell antitumor immunotherapy. Cancer Res.

[31] Berger C, Jensen MC, Lansdorp PM, Gough M, Elliott C, Riddell SR (2008). Adoptive transfer of effector CD8+ T cells derived from central memory cells establishes persistent T cell memory in primates. J Clin Invest.

